# Accuracy of volatile urine biomarkers for the detection and characterization of lung cancer

**DOI:** 10.1186/s12885-015-1996-0

**Published:** 2015-12-23

**Authors:** Peter J. Mazzone, Xiao-Feng Wang, Sung Lim, Humberto Choi, James Jett, Anil Vachani, Qi Zhang, Mary Beukemann, Meredith Seeley, Ray Martino, Paul Rhodes

**Affiliations:** Respiratory Institute, Cleveland Clinic, 9500 Euclid Ave., A90, Cleveland, OH 44195 USA; Metabolomx, Mountainview, CA USA; National Jewish Health, Denver, CO USA; University of Pennsylvania, Philadelphia, PA USA

**Keywords:** Volatile organic compounds, Urine, Biomarker, Lung cancer

## Abstract

**Background:**

The mixture of volatile organic compounds in the headspace gas of urine may be able to distinguish lung cancer patients from relevant control populations.

**Methods:**

Subjects with biopsy confirmed untreated lung cancer, and others at risk for developing lung cancer, provided a urine sample. A colorimetric sensor array was exposed to the headspace gas of neat and pre-treated urine samples. Random forest models were trained from the sensor output of 70 % of the study subjects and were tested against the remaining 30 %. Models were developed to separate cancer and cancer subgroups from control, and to characterize the cancer. An additional model was developed on the largest clinical subgroup.

**Results:**

90 subjects with lung cancer and 55 control subjects participated. The accuracies, reported as C-statistics, for models of cancer or cancer subgroups vs. control ranged from 0.795 – 0.917. A model of lung cancer vs. control built using only subjects from the largest available clinical subgroup (30 subjects) had a C-statistic of 0.970. Models developed and tested to characterize cancer histology, and to compare early to late stage cancer, had C-statistics of 0.849 and 0.922 respectively.

**Conclusions:**

The colorimetric sensor array signature of volatile organic compounds in the urine headspace may be capable of distinguishing lung cancer patients from clinically relevant controls. The incorporation of clinical phenotypes into the development of this biomarker may optimize its accuracy.

## Background

There has been a substantial amount of research in the field of molecular biomarker development aimed at improving our ability to predict who will develop lung cancer, detect lung cancer at an early stage, and characterize the cancer that is found. This work has most commonly used tissue or blood specimens to identify characteristic alterations in the genome, proteome, transcriptome, or metabolome of lung cancer patients.

Urine is a non-invasively collected biospecimen that has been relatively under-represented as a source of potential molecular biomarkers of lung cancer. Discovery level studies have identified differences in metal elements, [1] specific proteins, [2] proteomic signatures, [3] ratios of fluorescent peaks, [4] non-volatile metabolites, [5] exosomal proteins, [6] and tobacco metabolites, [7] in the urine of people with lung cancer.

Volatile organic compounds (VOCs) are present in very low concentrations in the headspace gas of urine samples. Over 700 VOCs have been identified in the urine of healthy volunteers. Diverse classes of VOCs are found in the urine including alcohols, aldehydes, amides, amines, carboxylic acids, esters, ethers, halides, heterocyclic compounds, hydrocarbons, ketones, nitriles, sulfides, terpenoids, and thiols [8]. There is a greater diversity of VOC classes in the urine than other biospecimen sources where VOCs can be measured, such as breath, skin, blood, and buccal mucosa [9]. These VOCs are felt to reflect metabolic alterations at the tissue level that enter the bloodstream and can leave the body in part by transfer into the urine. The composition of VOCs is affected by the altered metabolic properties of cancer cells, such as the manner in which they handle oxidative and energy stresses.

The premise that urine VOC profiles can be used to identify disease has been supported by studies of patients with celiac disease, [10] inflammatory bowel disease, [11] diabetes, [11] urinary tract infections, [12] and tuberculosis [13]. In addition, research aimed at developing forensics tools to identify individuals, and to locate people during disasters, has suggested unique patterns of VOCs are present in our urine [9, 14]. Discovery level studies have produced promising results for the identification of leukemia, colorectal cancer, and lymphoma through the use of gas chromatography–mass spectrometry analysis of the urine, [15] while bladder and prostate cancer studies have assessed VOC profiles detected by canine scent and ion mobility spectroscopy [16–18]. Two small discovery level studies using gas chromatography–mass spectrometry to detect a lung cancer signature from urine VOCs have been published, the first in mice injected with cancer cell lines, [19] and the second in humans .[20]. Promising results have encouraged us to explore this area further.

A colorimetric sensor array (CSA) is a cross-responsive chemical sensor whose output is a change in the color of its chemoresponsive elements upon exposure to VOCs [21]. The CSA signal is refined enough to separate VOCs by class and individually within a class when exposed to one VOC at a time, or to separate complex mixtures of VOCs from one another, such as those in the headspace gas of bacterial cultures [22]. In the current discovery level study we report on the accuracy of CSA derived signatures of the headspace gas of urine to detect and characterize lung cancer.

## Methods

This study was approved by the IRB of the Cleveland Clinic (CC) (IRB 1021). All study subjects signed informed consent.

Study subjects were included as cases if they had biopsy confirmed, untreated lung cancer or an imaging abnormality highly suspicious for lung cancer being scheduled for biopsy. Only those who later were biopsy confirmed remained cases in the study. Study subjects were included as controls if they were at risk for developing lung cancer based on age > 40 years and tobacco use of at least 10 pack-years, and/or a family history of lung cancer, and/or the presence of chronic obstructive pulmonary disease (COPD); or if they presented with an indeterminate lung nodule 8–30 mm in diameter that was ultimately confirmed to be benign based on biopsy or serial imaging. The duration of imaging was based on the size of the nodules as recommended in current guidelines [23]. Study subjects were excluded from participation if they had a prior history of lung cancer, a history of another cancer within 5 years, were receiving immunosuppression, or were using continuous supplemental oxygen. Consecutive subjects presenting to the outpatient Pulmonary department of the Cleveland Clinic, who met the above criteria, were approached. Approximately 50 % of those approached agreed to participate. Samples from all who agreed to participate were included in the analysis. Data collection included demographic variables and comorbidities for all subjects, nodule size for control subjects with lung nodules, and cancer histology and stage for the cancer subjects.

**Fig. 1:**
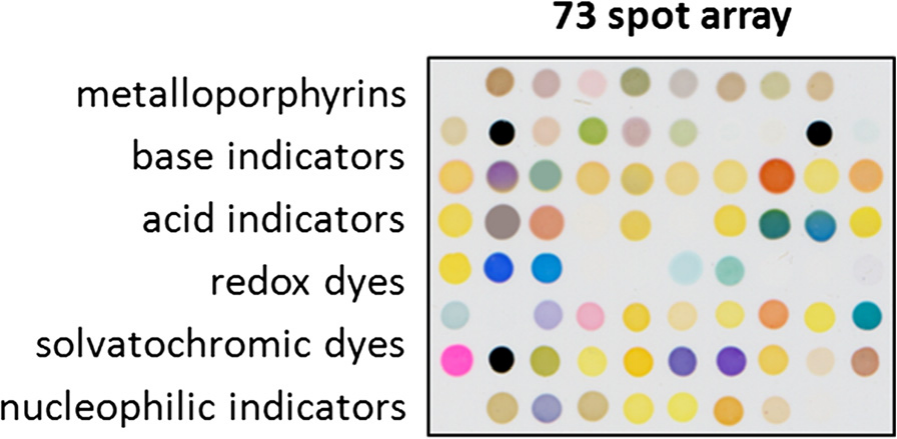
Sensor elements. The chemoresponsive elements include metalloporphyrins, base indicators, acid indicators, redox dyes, solvatochromic dyes, and nucleophilic indicators

The CSA was designed to have 73 chemoresponsive elements (Fig. 1). It was housed in a glass container above a sample of blotting paper to which the urine specimens were added. Study subjects provided a clean-catch urine sample at the end of a clinic visit in which they were at least 1 h from their last meal or drink. The sample was aliquoted and frozen at −80 °C within 2 h of being collected. At the time of testing the frozen urine was slowly thawed in a water bath then separated into four test conditions in order to maximize the sensor information: 1. Unaltered urine was analyzed with the CSA and separately used to measure the urine osmolality and perform a urine dipstick measurement, 2. A non-volatile acid (1 M tosic acid in a 1:1 volume ratio) was added in order to protonate organic acids to facilitate their evaporation, 3. A non-volatile base (1 M sodium hydroxide in a 1:1 volume ratio) was added to deprotonate amines, allowing them to evaporate more easily, 4. Urine was added to a pre-oxidation tube (sulfochromic acid on silica) to derivatize VOCs into more reactive species. Once prepared, 200 uL of each sample was added to a urine sensor cartridge which had been pre-warmed to 37 °C in an incubator for 20 min. An Epson V600 scanner imaged the sensor at 3 min intervals for 4 h with the cartridge held at 37 °C. Color difference maps were constructed by extracting the red, green, and blue values of the 73 indicators in the sensor array under each of the 4 conditions. The color vector of the initial image was subtracted from the color vectors of all subsequent images in order to construct a time series of color difference vectors. The person performing the urine tests (SL) was blinded to the study subject category (cancer or control).

Our statistical prediction model building procedure included four steps. The first step was feature extraction. To derive features that describe characteristics of the observed time series, a nonparametric local polynomial regression as well as a simple linear regression was produced from the data for each color time series. Four model-based features were derived for each time series: the area under the curve of the nonparametric regression; residual standard error of the fitted curve; total variation of the fitted curve (a statistical measure of variation of a nonlinear function); and the linear growth trend of the data (i.e. the slope of the linear regression line). The second step was feature filtering. The purpose of this step was to reduce variable dimension and select a set of relevant features for use in constructing an efficient prediction model. A univariate logistic regression was fit for each feature, and those whose C-statistic was greater than 0.6 were identified as potential predictor variables. The third step was model training. Our data set was randomly split into a training set and a testing set with 7:3 ratio. A variable selection procedure and correlation analysis were conducted to avoid multi-colinearity and overfit in the model. Clinical features known to be associated with lung cancer risk, including age, smoking history, and COPD, were included as variables. Random forest models were built using the subset of variables selected from the training set, with and without the inclusion of the clinical variables. The fourth step was model validation. The fitted random forests models were evaluated on the testing set. To avoid randomness in data split, we repeated the third and fourth steps 100 times and have summarized the prediction accuracy results. The prediction models (built from the training datasets) were applied to the subjects in the testing datasets. The observation in the testing data was classified as positive if the predictive probability of the outcome was greater than 0.5. The observation in the testing data was classified as negative if the predictive probability of the outcome was less than or equal to 0.5. For comparison, models were built in a similar fashion using only the clinical features and separately using all study subjects (rather than the 7:3 training:testing split).

Demographic variables were described using sample mean with standard deviation or proportion as appropriate. Categorical variables were compared using the Pearson’s chi-square test, and continuous variables were compared using the two sample independent *t*-test. All analyses were performed by using the R statistical package (www.r-project.org).

## Results

145 subjects were enrolled between 7/2012 and 3/2014, 90 with lung cancer and 55 controls. Control subjects were reported to have COPD more often than cancer subject (41.8 % vs. 23.3 %, *p* = 0.0188). There were no differences in other demographic variables or relevant comorbidities (Table 1). The control group included 31 at risk subjects and 24 who presented with indeterminate lung nodules. All demographic variables and relevant comorbidities were similar between these groups. The mean nodule diameter was 12.4 mm (range of 3–32). Of the 90 lung cancers, 6 were small cell, 53 adenocarcinoma, and 28 squamous cell carcinoma. There was a nearly equal distribution of localized and advanced stages of lung cancer (Table 2).

**Table 1:** Study Population

	Cancer (*N* = 90)	Control (*N* = 55)	Total (*N* = 145)	*p*-value
Age – Mean (SD)	67.3 (10.3)	64.6 (9.2)	66.3 (10.0)	0.1198
PackYears – Mean (SD)	42.3 (28.0)	38.1 (28.1)	40.7 (28.0)	0.2868
Sex				0.3838
Female	41 (45.6 %)	21 (38.2 %)	62 (44.1 %)	
Male	49 (54.4 %)	34 (61.8 %)	83 (55.9 %)	
Smoking History				0.6401
Current	19 (21.1 %)	15 (27.3 %)	34 (23.4 %)	
Former	64 (71.1 %)	37 (67.3 %)	101 (69.7 %)	
Never	7 (7.8 %)	3 (5.5 %)	10 (6.9 %)	
COPD	21 (23.3 %)	23 (41.8 %)	44 (30.3 %)	0.0188
DM	10 (11.1 %)	7 (12.7 %)	17 (11.7 %)	0.7691
Elevated Cholesterol	22 (24.4 %)	14 (25.5 %)	36 (24.8 %)	0.8913

**Table 2:** Lung cancers

	Stage I	Stage II	Stage III	Stage IV	Unclear	Total
Adenocarcinoma	14	6	18	12	3	53
Squamous	11	5	8	3	1	28
Other NSCLC	1	1	0	1	0	3
Small cell	0	1	3	2	0	6
Total	26	13	29	18	4	90

Models were developed and tested comparing cancer and histology subgroups to controls. The accuracies, reported as C-statistics, ranged from 0.795 – 0.917. Models built from the entire dataset had similar accuracies (C-statistic 0.792 - 0.923). The accuracies were higher when the histology subgroups were compared to controls. There was little difference in the model accuracies when urine features alone were used to develop the models compared to models that included clinical variables. The model accuracies of stage I cancers vs. controls were equally, or more, accurate though the numbers of subjects with stage I were relatively small. Models developed and tested to characterize cancer histology, and to compare early to late stage cancer, were very accurate (Table 3). Normalization of the data for urine osmolarity and specific gravity did not substantially influence model accuracies. Models developed using clinical variables only were less accurate (C-statistics 0.543 – 0.687).

**Table 3:** Accuracy of models: Validated C-statistics with confidence intervals through model training on 70 % of subjects and testing on 30 %

	Urine Only	Urine + Clinical	Sensitivity (%)	Specificity (%)
All cancer vs. control	0.795 (0.768-0.823)	0.810 (0.782-0.838)	81.4 (77.9-84.9)	60.0 (56.9-63.1)
NSCLC vs. control	0.804 (0.783-0.825)	0.809 (0.782-0.836)	80.0 (76.8-83.2)	64.7 (60.8-68.6)
Adenocarcinoma vs. control	0.900 (0.879-0.920)	0.917 (0.899-0.936)	73.3 (69.1-77.6)	89.7 (87.6-91.8)
Squamous vs. control	0.821 (0.797-0.847)	0.821 (0.790-0.853)	60.0 (55.3-64.7)	87.9 (84.4-91.4)
Stage I vs. control	0.873 (0.848-0.90)	0.875 (0.856-0.894)	49.3 (44.9-53.7)	92.8 (89.5-96.1)
Stage I Adenocarcinoma vs. control	0.875 (0.843-0.906)	0.876 (0.846-0.906)	36.0 (27.7-44.3)	96.9 (95.2-98.6)
Stage I Squamous vs. control	0.940 (0.925-0.955)	0.938 (0.923-0.953)	46.7 (39.4-53.9)	97.6 (96.5-98.7)
Adenocarcinoma vs. Squamous	0.849 (0.828-0.87)	0.859 (0.831-0.887)	84.0 (80.8-87.3)	72.7 (67.2-78.2)
Stage I vs. IV	0.922 (0.892-0.952)	0.922 (0.892-0.952)	92.7 (90.2-95.2)	68.0 (60.3-75.7)
All cancer vs. control (matched)	0.968 (0.954-0.982)	0.970 (0.954-0.986)	95.5 (92.4-98.6)	88.0 (81.2-94.8)
NSCLC vs. control (matched)	0.977 (0.957-0.997)	0.987 (0.977-0.997)	86.7 (83.6-89.8)	96.0 (92.8-99.2)

To assess the influence of the subjects’ phenotypes on model accuracy we performed additional analyses. The study population was divided by sex, age (<55, 55–70, >70), and COPD into 12 subgroups. The largest subgroup (male, age 55–70, without COPD) contained 30 subjects (18 cancer, 12 control). Models developed and tested within this phenotype were very accurate, with a C-statistic of 0.970 for all cancer vs. control, and 0.987 for non-small cell carcinoma vs. control (Table 3).

## Discussion

We report the results of the development of a CSA based profile of urine headspace gas VOCs as a biomarker that could assist with the diagnosis and characterization of lung cancer. To our knowledge, this is the first study using a cross-responsive chemical sensor for this purpose. We found the CSA profile had good accuracy at separating subjects with lung cancer from clinically appropriate controls; that the accuracy improved when subtypes of lung cancer were compared to controls; and that the accuracy was very high when the signatures were developed within a specific subset of subjects defined by their clinical phenotype. Finally, the results showed promise at being able to characterize the lung cancer’s histology and stage.

The current report describes a discovery level study of a novel urine based lung cancer biomarker. To advance this work, technical validation of the test and clinical validation of the results will be required. Technical validation will include the development of standard operating procedures for urine collection and processing, and confirmation of uniform performance of the CSA from one batch of sensors to the next. Relatively little is known about the proper conditions in which urine should be collected and processed for VOC evaluation. Studies have suggested each individual’s urine VOC signature is unique, with a small amount of variability based on diet which is exceeded by the variability between individuals [24, 25]. Storage of urine samples for up to 1 month at −80 °C seems to have little influence on the urine VOC profile, [13] whereas storage at room temperature for 3 days may influence the concentration of VOCs identified [26]. The number and classes of VOCs detected is highest in acidified and basified urine, [13, 26] with only a small number of VOCs being ubiquitous independent of pH [8]. Other components of urine dipstick measurements did not affect classification accuracy in a canine bladder cancer study [16].

Additives were used to maximize the liberation of VOCs based on pH and oxidation. Urine samples were processed and frozen within 2 h but were tested at a variable distance from the time of processing (some over 1 year later). There did not appear to be any impact from normalization of results for urine concentration measures. As a next step, we will learn more about the influence of diet, the type of collection and storage containers, the time to processing and testing, the ideal additives, the optimal urine volume, temperature during testing, and the need for normalization to other urine values.

Lung cancer is heterogeneous in its clinical presentation and molecular makeup, as is the group of people in whom it develops. It is likely that one metabolic biomarker cannot accurately identify all patients with lung cancer. A patient’s clinical phenotype could influence the metabolic baseline. Alterations from this baseline may be useful in distinguishing a non-cancer from a cancer biosignature. Our exploratory results support very high accuracy of the metabolic biosignature when developed within a relatively uniform clinical phenotype. Clinical validation of a technically validated sensor platform will require a larger number of subjects in each clinical phenotype to be confident in the accuracies reported.

The output of the CSA, a cross-responsive sensor, is influenced by the mixture of VOCs to which it is exposed. The output is not able to identify the components of this mixture. Gas chromatography–mass spectrometry has been used in a small study to try to define the individual VOCs that make up the mixture. Further work in this area will help us understand the nature of the VOC signatures. Sensor technologies are more apt to be useful in the clinical setting because they are inexpensive and less technically demanding to apply and interpret.

Other limitations of our study include the small sample size for some of the comparisons where the accuracy was highest. These comparisons should be viewed as exploratory, helping to guide the next phase of urine biomarker development. It is not clear that the urine processing methodologies used in this study are optimal, and minor inconsistencies in the sensor manufacturing could impart unseen biases in the results. These issues will need to be addressed as part of the validation of this biomarker for clinical use. The distinguishing signatures from a technically validated instrument will then require validation on an independent cohort of a relevant population. The target for this test could be an upfront screening test or an adjunct to nodule evaluation. The validation cohort will need to reflect these targets. The results presented compare favorably with other biomarkers of early detection and/or nodule management.

## Conclusions

In conclusion, the CSA signature of urine headspace gas VOCs is capable of distinguishing cancer patients from clinically relevant controls. The incorporation of clinical phenotypes into the development of this biomarker may optimize its accuracy.

## Abbreviations

CC: Cleveland Clinic
COPD: Chronic obstructive pulmonary disease
CSA: Colorimetric sensor array
VOC: Volatile organic compound
